# Impact of Vimentin on Regulation of Cell Signaling and Matrix Remodeling

**DOI:** 10.3389/fcell.2022.869069

**Published:** 2022-03-11

**Authors:** Zofia Ostrowska-Podhorodecka, Isabel Ding, Masoud Norouzi, Christopher A. McCulloch

**Affiliations:** Faculty of Dentistry, University of Toronto, Toronto, ON, Canada

**Keywords:** EMT, vimentin, PTM, cell migration, cell adhesion

## Abstract

Vimentin expression contributes to cellular mechanoprotection and is a widely recognized marker of fibroblasts and of epithelial-mesenchymal transition. But it is not understood how vimentin affects signaling that controls cell migration and extracellular matrix (ECM) remodeling. Recent data indicate that vimentin controls collagen deposition and ECM structure by regulating contractile force application to the ECM and through post-transcriptional regulation of ECM related genes. Binding of cells to the ECM promotes the association of vimentin with cytoplasmic domains of adhesion receptors such as integrins. After initial adhesion, cell-generated, myosin-dependent forces and signals that impact vimentin structure can affect cell migration. Post-translational modifications of vimentin determine its adaptor functions, including binding to cell adhesion proteins like paxillin and talin. Accordingly, vimentin regulates the growth, maturation and adhesive strength of integrin-dependent adhesions, which enables cells to tune their attachment to collagen, regulate the formation of cell extensions and control cell migration through connective tissues. Thus, vimentin tunes signaling cascades that regulate cell migration and ECM remodeling. Here we consider how specific properties of vimentin serve to control cell attachment to the underlying ECM and to regulate mesenchymal cell migration and remodeling of the ECM by resident fibroblasts.

## Introduction

Phenotypic regulation in many cell types is influenced by their interactions with the extracellular matrix (ECM), a major structural component of many organs and soft connective tissues that is comprised mainly of fibrillar collagens (Begum et al., 2017; Liu et al., 2018; Rada et al., 2018), glycoproteins such as fibronectin (Kendall and Feghali-Bostwick, 2014; Li C.-L. et al., 2017; Yu et al., 2018) and vitronectin (Braam et al., 2008; Hurt et al., 2010) and a broad repertoire of proteoglycans (Teng et al., 2012; Sun et al., 2017; Cao et al., 2018) and polysaccharides (An and Brodsky, 2016). In many organs the ECM is a highly dynamic structure and, in some tissues, ECM proteins like collagen undergo surprisingly rapid physiological turnover (Sodek, 1977). Remodeling involves deposition, degradation, and modifications of the ECM by resident cells and secreted enzymes (Kim et al., 2011; Lu et al., 2011; Lu et al., 2012; Lee et al., 2019; Nallanthighal et al., 2019). ECM remodeling plays a central role in tissue and organ health and is intimately involved in the migration of cells that occurs in developmental processes, wound healing and cancer metastasis.

The physical reorganization of fibrillar proteins that accompanies the migration of fibroblasts through the ECM is a central feature of collagen remodeling (Feng et al., 2014), which is crucial for the maintenance of tissue health in many organs (Cox and Erler, 2011). After an injury or chronic infection, tissues often exhibit a short-term wound healing response that is intended to create a new and functionally appropriate ECM that then enables restoration of tissue structure (Walker et al., 2018). The mechanical properties of ECM, such as the stiffness of collagen fibrils or of the underlying substrate to which cells are attached, play crucial roles in processes such as epithelial-mesenchymal transition (EMT) and cell differentiation (Engler et al., 2006; Saha et al., 2008; Santiago et al., 2010). Epithelial-mesenchymal transition (EMT) is a potentially reversible process by which epithelial cells transdifferentiate into highly motile cells with mesenchymal cell phenotypes. During EMT, epithelial cells undergo modifications that affect the structure of intercellular junctions and of adhesion complexes with the ECM that affect ECM remodeling. Modifications associated with EMT also include the disaggregation of epithelial cells from one another and the underlying basement membrane. Subsequently, new transcriptional programs are activated that promote the acquisition of mesenchymal characteristics in affected cells (Thiery and Sleeman, 2006; Thiery et al., 2009; Nieto et al., 2016).

As described in the Human Protein Atlas database (Atlas, 2021), vimentin intermediate filaments (VIFs) are expressed in a wide variety of tissues including skin, kidney, and lung (Schaffeld et al., 2001; Mendez et al., 2010; Lowery et al., 2015; Uhlen et al., 2015). Vimentin is a 54 kDa, 466 amino acid Type III intermediate filament (UniProtKB-P08670). Vimentin exhibits a tripartite structure consisting of a central α-helical “rod” domain, flanked by intrinsically disordered amino-terminal “head” and carboxy-terminal “Tail” domains (Strelkov et al., 2001). In physiological conditions, vimentin spontaneously assembles into 10 nm diameter filaments (Herrmann and Aebi, 1998). Filament assembly is initiated from elementary, parallel coiled-coil α-helical dimeric building blocks, which self-associate in a half-staggered, anti-parallel manner to yield tetramers (Chernyatina et al., 2015). Subsequently, lateral association of 8 tetrameric subunits results in unit-length filaments, which longitudinally extend to form mature vimentin filaments (Herrmann and Aebi, 1998), which is considered in more detail in earlier reviews (Danielsson et al., 2018) (Ostrowska-Podhorodecka and McCulloch, 2021).

The recent uptick of interest in vimentin originates in part from the growing appreciation of its diverse roles in a broad range of cellular functions that affect tissue and organ structure. Notably, alterations of vimentin expression are linked to diseases including lung and liver fibrosis and several types of cancer (Li F. J. et al., 2017; Battaglia et al., 2018; Strouhalova et al., 2020), all of which involve cell migration and ECM remodeling. In addition to these discrete pathological conditions, vimentin expression is crucial for effective wound healing and tissue regeneration. Higher vimentin expression is associated with enhanced cell motility, adhesion to the ECM and collagen deposition (dos Santos et al., 2015; Cheng et al., 2016). Indeed, the importance of vimentin is now widely recognized in cellular functions ranging from motility to signal transduction (Mendez et al., 2010; Lowery et al., 2015). In contrast, the lack of vimentin in vimentin knockout mice resulted in the loss of cell morphology and reduced cell adhesion, as well as impairment in the directional migration of fibroblasts. In addition, at the tissue level, vimentin deficiency reduced the capacity for wound-healing (Blanchoin et al., 2014; Danielsson et al., 2018). For more detailed information on this topic, see reviews of vimentin functions in matrix adhesion (Danielsson et al., 2018; Ostrowska-Podhorodecka and McCulloch, 2021).

Expression of vimentin in epithelial cells during EMT is associated with the adoption of a more mesenchymal cell shape, increased focal adhesion formation and enhanced cell motility (Lepekhin et al., 2001; Mendez et al., 2010; Ostrowska-Podhorodecka et al., 2021). Conversely, diminished vimentin expression in mesenchymal cells is associated with reduced motility and the adoption of an epithelial cell like shape (Mendez et al., 2010; Ostrowska-Podhorodecka et al., 2021). While it has been suggested that vimentin expression affects ECM remodeling and cell migration through the ECM (Mendez et al., 2010; Nieto et al., 2016; Cheng and Eriksson, 2017; Patteson et al., 2019; Ding et al., 2020; Ostrowska-Podhorodecka et al., 2021), the definitive roles played by vimentin in regulating ECM structure (Menko et al., 2014; Walker et al., 2018), autophagy (Su et al., 2019), mRNA processing (Challa and Stefanovic, 2011) and transcriptional regulation (Deng et al., 2013) remain elusive. Through its integration of environmental signals, vimentin seems to adjust the dynamics and structures of the microtubule and actomyosin networks, which are crucial for generating the forces needed for cell migration (Battaglia et al., 2018). As the regulatory functions of vimentin in cell migration and ECM remodeling are not well-understood, we consider a potential role for vimentin in integrating signaling, matrix remodeling and migration.

## Cellular Localization of Vimentin Intermediate Filaments

**FIGURE 1:**
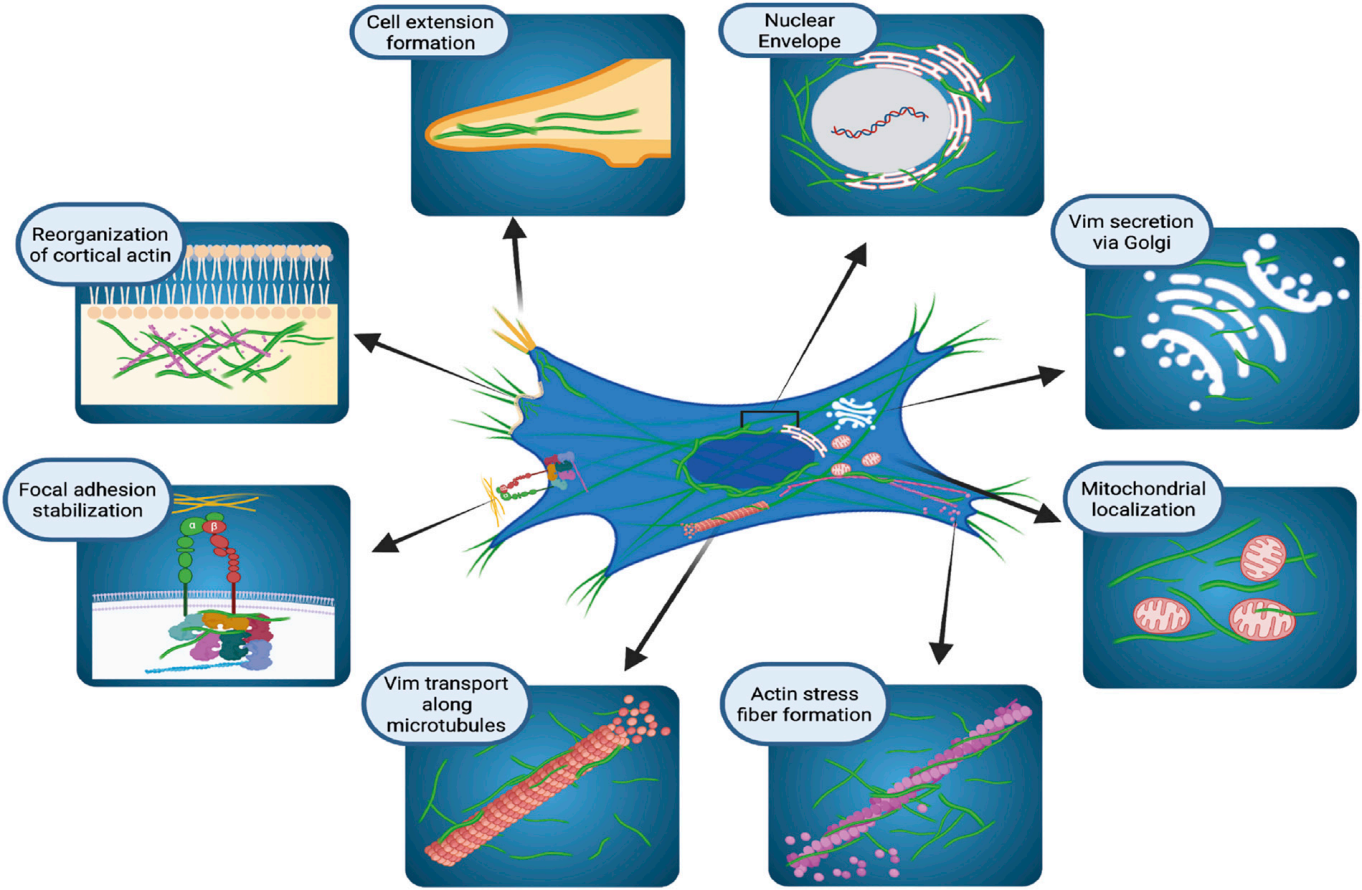
Cellular localization and functions of vimentin intermediate filaments. Schematic illustration of how vimentin controls several, diverse cellular functions in organelle anchoring, cytoskeletal plasticity, focal adhesion regulation, and cell migration. Figure created with BioRender.com.

For many years VIFs were considered as very stable cytoskeletal structures whose principal functions provided resistance to mechanical stress (Kim and Coulombe, 2007) and participation in mechanotransduction (Gregor et al., 2014). More recent evidence indicates that the vimentin network exhibits a broad array of properties that support essential cellular functions (Duarte et al., 2019; Patteson et al., 2019; Strouhalova et al., 2020). In this context, vimentin is localized to discrete cytoplasmic and membrane compartments (Figure 1) in mesenchymal cells (Mendez et al., 2010), fibroblasts (Mendez et al., 2010; Helfand et al., 2011; Ding et al., 2020), astrocytes (Lepekhin et al., 2001), epithelial cells (Vuoriluoto et al., 2011), cells in lymphoid tissues (Otsuki et al., 2011), glandular cells (Peuhu et al., 2017), and various cancer cell types (Vuoriluoto et al., 2011; Havel et al., 2015; Rawla et al., 2019; Kuppe et al., 2021; Thalla et al., 2021). In the cell body, vimentin is predominantly perinuclear (Dupin et al., 2011) where it protects DNA from mechanical damage (Patteson et al., 2019), but vimentin also co-localizes with the endoplasmic reticulum (Lee et al., 2020) and contributes to the positioning of mitochondria and Golgi apparatus in the cytosol (Gao and Sztul, 2001; Lowery et al., 2015), indicating that vimentin may play a broader role in cell regulation than previously recognized.

The presence of VIFs in the cell periphery during mitosis is associated with the organization of cortical actin filament arrays, suggesting that vimentin filaments may help to strengthen the cortex during cell division (Duarte et al., 2019). Electron microscopy and live-cell imaging of cultured cells shows that vimentin filaments undergo continuous and relatively dynamic changes of assembly and localisation to produce a broad repertoire of VIF structures (Herrmann et al., 1996; Noding et al., 2014; Lowery et al., 2015; Strouhalova et al., 2020). These structures range from flexible, extended polymerized networks to non-filamentous structures that are observed in particles of various sizes (Chou et al., 1990; Robert et al., 2015; Premchandar et al., 2016). In cultured fibroblasts, VIFs are rather homogenously distributed throughout the cytoplasm. But fibroblasts exhibit a perinuclear location of VIF after treatment with agents that affect vimentin filament organization, such as withaferin A (Ding et al., 2020) or the p21 kinase inhibitor, IPA3 (Ostrowska-Podhorodecka et al., 2021). The dynamic assembly and disassembly of VIFs helps cells to adapt to heat-shock or oxidative stress (Perez-Sala et al., 2015; Robert et al., 2015; Duarte et al., 2019). The rapid and reversible remodeling of VIFs relies on the exchange of subunits and on post-translational modifications, which we consider later in this review.

## Role of Vimentin as an Effector and a Target of Post-Transcriptional Gene Regulation in ECM Biology

**FIGURE 2:**
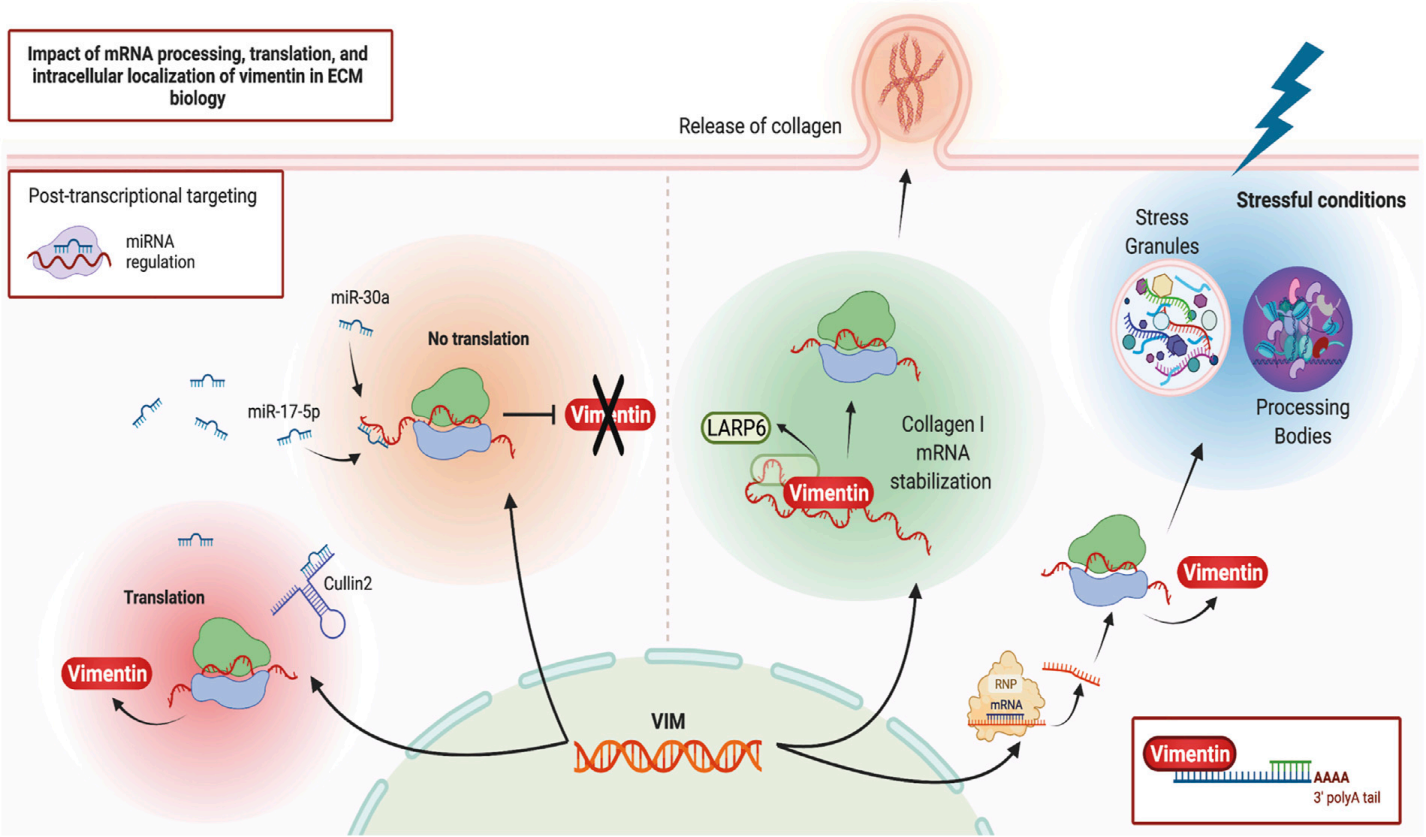
Vimentin and post-transcriptional gene regulation. Schematic illustration of various post-transcriptional regulatory pathways that can impact or be impacted by vimentin. Figure created with BioRender.com.

Vimentin may regulate ECM remodeling through its impact on post-transcriptional gene regulation, which in turn impacts the synthesis and degradation of ECM proteins. One of the regulatory processes affected by vimentin is the spatial regulation of RNA expression and its interaction with ribonucleoprotein (RNP) complexes (Figure 2). Ultrastructural *in situ* hybridization experiments demonstrate that ∼29% of total cytoplasmic poly (A) mRNAs co-localize with vimentin filaments (Bassell et al., 1994). More direct evidence of vimentin’s involvement in post-transcriptional gene regulation arises from its interaction with type I collagen mRNAs (Zhang and Stefanovic, 2016). Specifically, RNA co-purification and *in situ* hybridization experiments demonstrate a tripartite assembly between the 5′ untranslated region stem-loop (5′ SL) domain of collagen mRNAs, La ribonucleoprotein domain family member 6 (LARP6) protein, and vimentin filaments (Cai et al., 2010; Challa and Stefanovic, 2011). In this instance, vimentin regulates collagen synthesis by sequestering LARP6-bound collagen mRNAs. During wound healing or in fibrotic lesions, when demand for type I collagen is increased (Cheng and Eriksson, 2017), these mRNAs are made available for translation (Challa and Stefanovic, 2011). In addition to collagen mRNA, vimentin can specifically bind and post-transcriptionally regulate several other genes, including binding to the 5′ UTR and repression of the mu opioid receptor mRNA in Mouse neuroblastoma cell lines (Song et al., 2013), binding to the 3′ UTR and stabilisation of the alkaline phosphatase mRNA in human primary osteoblasts (Schmidt et al., 2015), and binding to the 3′ UTR and stabilisation of the tissue factor (TF) mRNA in human breast cancer cells through blocking miR-dependent negative regulation of TF mRNA (Francart et al., 2020). Currently, collagen is the only ECM component whose mRNA is post-transcriptionally regulated by vimentin.

More recently, proximity-based assays have shed light on vimentin’s involvement in cellular differentiation, homeostasis, and stress response through its association with RNA-binding proteins, misfolded aggregates, Stress Granules and Processing Bodies (Lin et al., 2016; Pattabiraman et al., 2020). These processes are of particular importance for cells subjected to repeated mechanical or inflammatory stressors. Stress Granules and Processing Bodies are cytoplasmic compartments comprised of translationally repressed mRNAs, post-transcriptional regulatory factors, and other RNA binding proteins. These membrane-less organelles form part of the cellular response to a broad range of stressful conditions such as starvation and protein misfolding. For example, these responses prioritize the translation of stress response mRNAs and targeting the mRNAs codifying misfolded proteins for degradation (Luo et al., 2018; Marcelo et al., 2021). Accordingly, during differentiation and under stressful conditions, vimentin protects cells and their progeny by spatially segregating misfolded proteins, Stress Granules and other cytoplasmic RNP complexes. Vimentin directs their asymmetric partitioning during mitosis so that undesirable metabolites accumulate in one daughter cell while the other daughter cell remains healthy (Ogrodnik et al., 2014; Pattabiraman et al., 2020). The precise mechanisms and extent of vimentin’s involvement in cellular differentiation, stress response and the resultant modifications of ECM remodeling through post-transcriptional gene regulation are not understood in depth but almost certainly will provide useful avenues for future research in IF biology.

Vimentin is one of the main mediators of EMT and metastasis in a variety of cancers (Satelli and Li, 2011; Strouhalova et al., 2020) and is itself a target of post-transcriptional gene regulation. One well-studied pathway that affects vimentin expression at the transcript level is through MicroRNAs (miRNAs) (Guo et al., 2014), which are ∼22 nucleotide-long non-coding RNAs that bind to the 3′ untranslated region (UTR) of target mRNAs and mark them for translational repression (Bartel, 2004). For instance, through direct interaction with the 3’ UTR of vimentin mRNA and its subsequent downregulation, miR-30a supresses the invasive phenotypes of breast cancer cell lines (Cheng et al., 2012) while miR-17-5p inhibits the metastasis of colorectal cancer in liver tissues (Kim et al., 2020). Conversely, in an alternative mechanism, a non-coding RNA “sponge” known as Cullin2 circular RNA (or circ-10720) regulates vimentin expression by sequestering vimentin-targeting miRNAs, thus promoting EMT (Meng et al., 2018). Therefore, vimentin mRNA is a target of post-transcriptional regulatory events that impact the abundance of vimentin protein. Vimentin protein also contributes to post-transcriptional regulation of other mRNAs, especially during cell differentiation and environmental stress. Obtaining a more detailed understanding of these post-transcriptional regulatory networks could impact future translational research as these networks could provide potential targets for drug development.

## Viscoelastic Properties of Vimentin Enhance Cell Resistance to Deformation

The migration of cells through soft connective tissues depends in part on their ability to remodel the ECM through synthesis and degradation. Migration in turn is reliant on the ability of cells to attach to ECM proteins and to navigate through pores in the ECM, which, depending on their size, may require cell deformation. The deformability of a cell and its ability to return to its original shape are affected by the viscoelastic properties of cells and their constituents (Moeendarbary et al., 2013; van Bodegraven and Etienne-Manneville, 2021). VIFs exhibit distinct viscoelastic properties that are not typically exhibited by other filamentous biopolymers such as actin filaments (Shah et al., 1998). This unique trait confers distinct rheological properties upon vimentin filaments. As a result, vimentin not only enhances cell integrity after exposure to shear force, but also provides flexibility in cells recovering from membrane deformation. The fine tuning of membrane stiffness by vimentin may contribute to a wide array of biological functions including cell migration, division, cell adhesion to substrates, autophagy, and signal transduction (Wang and Stamenovic, 2002; Vakhrusheva et al., 2019).

Cell migration through ECMs like collagen networks often provokes nuclear rupture, which is related to constriction and deformation of the nuclear membrane as cells traverse pores in the ECM. VIFs form intricate networks such as the nuclear cage that extends from the perinuclear region to the sub-cortex. In these networks, vimentin filaments function as elastic springs, dissipating tensile forces when migrating cells squeeze through constricting pores. These properties of vimentin can prevent extensive fluctuations of nuclear shape (Block et al., 2018). In addition, vimentin may interact with nucleases to buffer DNA damage (Irianto et al., 2016). In view of these findings, vimentin may play a crucial role in maintaining the precision of signaling by safeguarding DNA integrity, which is observed in cells subjected to migration-induced nuclear deformation (Patteson et al., 2019). Taken together, VIF expression contributes to cell viability by limiting organellar deformation during mechanical stress and by facilitating the recovery of cell and organellar shape.

## Post-Translational Modifications of Vimentin Affect Migration and the ECM

**FIGURE 3:**
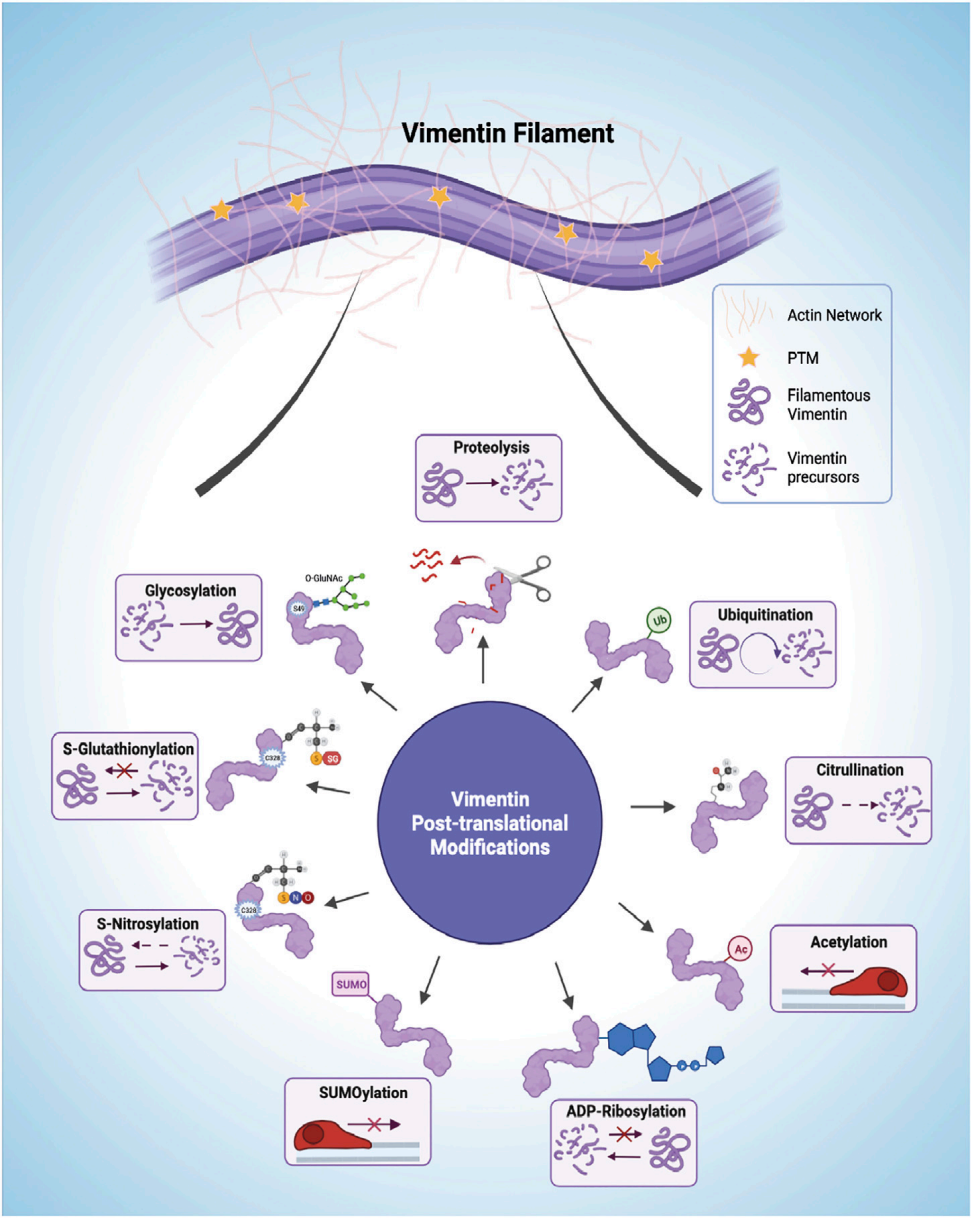
Post-translational modifications (PTMs) of vimentin intermediate filaments (VIFs). VIFs undergo multiple PTMs, including proteolysis, ubiquitination, citrullination, acetylation, ADP-Ribosylation, SUMOylation (SUMO), S-Nitrosylation, S-Glutathionylation, and glycosylation. The horizontal arrows presented in the acetylation and SUMOylation panel shows decreased cell mobility. Figure created with BioRender.com.

Post-translational modifications (PTMs) are implicated in the spatiotemporal regulation of vimentin expression and organization of the VIF network, which consequently impacts the stability of the underlying cytoskeleton and remodelling of the ECM (Kalyanasundaram et al., 2021). Currently it is not straightforward to dissect the repertoire of signaling pathways associated with each vimentin PTM because of the sheer redundancy and complexity of the system and its multiple components (Figure 3). Moreover, modifications to single amino acids in vimentin may yield opposite downstream effects compared with modifications to multiple residues because of altered binding of interacting proteins and the resultant generation of downstream signals. One of the most carefully studied PTMs of vimentin is phosphorylation, which can promote VIF disassembly into squiggles, as seen in migrating cells (Yang et al., 2019). Phosphorylation is one of the cardinal features of signaling processes that are contemporaneously stimulated by other PTMs (Snider and Omary, 2014). The phosphorylation and dephosphorylation of vimentin have been reviewed previously. Shi et al., 2016 provide a comprehensive analysis of the effect of vimentin phosphorylation on cell motility. Accordingly, we focus here on separate vimentin PTMs that are associated with cell migration and/or ECM remodeling.

### Proteolysis

Vimentin, like other intracellular proteins, is prone to caspase-mediated proteolytic cleavage. Phosphorylation protects vimentin from caspase-mediated proteolysis (Tripathi and Kulkarni, 2021), which occurs at vimentin D85 (via caspase 3 and caspase 7) and D259 (via caspase 6) to generate pro-apoptotic N-terminal fragments (Byun et al., 2001). AKT1 activation induces migration of sarcoma cells through an interaction with the vimentin head region, resulting in S39 phosphorylation and protection from caspase-induced proteolysis of vimentin (Zhu et al., 2011), thereby providing an example of the central position of vimentin in signaling systems that affect cell migration.

### Ubiquitination

Proteasome-dependent degradation of vimentin by ubiquitination promotes the collapse of vimentin network architecture, triggering major cytoskeletal rearrangements. Specific ubiquitination sites are not well studied and recorded. Vimentin can be ubiquitinated at K97, K120, K129, K139, K143, K168, K188, K223, K236, K282, K294, K313, K334, K373, K439, and K445 (Database, 2022). Substrate proteins are linked to ubiquitin *via* distinct ubiquitin lysine residues (K6, K11, K27, K29, K33, K48, and K63) (Technology, 2022). Beclin 1, a critical regulator of autophagy, increases cell migration by interacting with vimentin to affect its K48-linked ubiquitination. The K48-linked polyubiquitin chains mainly target proteins for proteasomal degradation (Manohar et al., 2019). Beclin 1 also interacts with ubiquitin-specific peptidase 14, a key de-ubiquitinase of vimentin (Cheng et al., 2019). Thus, autophagy processes mediated through ubiquitination, which impact vimentin filament integrity, affecting cell migration. Moreover, vimentin ubiquitination is balanced by deubiquitination in selected systems. For instance, in human gastric cancer cell lines, the deubiquitinating enzyme USP14 directly interacts with vimentin and stabilizes it through deubiquitination (Zhu et al., 2017). This report also shows that miR320, as a tumor suppressor, is upstream of U14 and vimentin. It downregulates vimentin directly by targeting its 3′UTR, or indirectly by inhibiting the USP14 deubiquitination pathway (Zhu et al., 2017).

### Citrullination

The search for causative mechanisms in rheumatic diseases and fibrosis, which involves extensive and often dysregulated ECM remodeling, has generated considerable interest in citrullinated vimentin, which involves the conversion of arginine residues to citrulline by the enzyme, peptidyl arginine deiminase (PAD) (Pruitt et al., 2014). Vimentin is a substrate for PAD2 that citrullinates residues in the non-α-helical head domain, which contains about 9% arginine residues (Inagaki et al., 1989; Hsu et al., 2014). A vimentin peptide with citrullinated R176, which is found in coil 1B of vimentin, was identified in a vimentin pool secreted from lung macrophages and characterized by tandem MS2 (Li et al., 2021). Vimentin can also be citrullinated at several arginine residues in the tail domain. For example, R440 and R450 are citrullinated in reactive astrocytes in brain tissues of scrapie-infected mice (Jang et al., 2020). Citrullination impairs vimentin filament assembly, which enhances the formation of soluble precursors that are transported extracellularly (Inagaki et al., 1989) and that subsequently elicit autoimmune responses in joints affected by rheumatoid arthritis (Musaelyan et al., 2018) and in liver fibrosis (Vassiliadis et al., 2012). Citrullinated vimentin is increased after cell injury and is strongly expressed in the leading edge of repair-modulating leader cells, which stimulates their migration and differentiation into myofibroblasts (Walker et al., 2018). These leader cells display invasive potential that facilitates three-dimensional migration (Bleaken et al., 2016) and contribute to alterations of ECM structure.

### Acetylation

The acetylation of vimentin and other EMT-related proteins affects the migratory capacity and the metastatic properties of various types of cancer cells (Boggs et al., 2015). Acetylation mainly targets vimentin at lysine residues, including K294, K313, K334, K373, and K439 (Yang et al., 2019). Hyperacetylation is generally associated with decreased cell motility through enhancement of vimentin filament stability and the formation of EMT-related protein complexes (Yang et al., 2019). In contrast, deacetylation of K120 of vimentin reduces metastasis in hepatocellular carcinoma, suggesting that acetyl-modified K120 regulates cell migration, possibly through upregulation of Snail and downregulation of E-cadherin, processes that ultimately enhance EMT (Guo et al., 2018).

### ADP-Ribosylation

ADP-ribosylation is exhibited by certain pathogenic bacterial species and involves the attachment of ADP-ribose to host proteins through the formation of an O-glycosidic bond by pathogen-derived enzymes (Palazzo et al., 2018). Vimentin in mammalian cells is a target of *Streptococcus pyogenes* (Icenogle et al., 2012), which involves the secretion of the exotoxin, ADP-ribosyltransferase and is followed by sharp reductions of host cell migration, alterations of ECM structure and increased spreading of the pathogen (Coye and Collins, 2004).

### SUMOylation

The conjugation of small, ubiquitin-like modifier (SUMO) protein to lysine residues of acceptor proteins like vimentin can impact cell migration through poorly defined mechanisms (Wang et al., 2010). PIAS1 mediates SUMOylation of vimentin (K439, K445) in the C-terminus, which disrupts filament disassembly (Li et al., 2020). This modification increases vimentin solubility by inducing hyperphosphorylation of the vimentin N-terminus and retards cell migration, suggesting that vimentin filament assembly is required for efficient cell migration.

### S-Nitrosylation

Reactive nitric oxide transfers nitrosyl moieties from donor to acceptor proteins, a process that involves nitric oxide synthase and modifies C328 of vimentin in response to mechanotransduction through the Akt pathway (Huang et al., 2009) and in stress sensing (Perez-Sala et al., 2015). The presence of thiol modifications seems to retard the longitudinal assembly of vimentin filaments without inhibiting the formation of more mature filaments (Kaus-Drobek et al., 2020) and leads to altered ECM remodeling. As altered network rearrangements, filament stabilization, and bundling are linked to cysteine modifications of vimentin structure (Viedma-Poyatos et al., 2020), it will be important to define how these modifications contribute to altered cell migration (Kaschula et al., 2019) and potentially, ECM remodeling.

### S-Glutathionylation

Modifications of vimentin C328 protect against electrophilic and oxidative stress by preserving the flexibility of VIFs (Perez-Sala et al., 2015). But unlike S-nitrosylation, S-glutathiolylation completely blocks the maturation of unit length filaments to mature filaments (Kaus-Drobek et al., 2020), indicating that this process is an efficient molecular switch that contributes to the assembly of the vimentin network, thereby affecting cell-mediated remodeling of the ECM.

### Glycosylation

This vimentin PTM is mediated by the addition of O-linked β-N-acetylglucosamine (O-GlcNAc) on serine and threonine residues mediated by O-GlcNAc transferase and O-GlcNAcase (Hanover et al., 2010; Hart et al., 2011; Hart, 2014). Glycosylation of vimentin is restricted to the head domain of mature filaments (i.e., not ULFs) at residues T33, S34, S39, and S49, which impacts the formation of homo-oligomeric complexes between adjacent filaments (Tarbet et al., 2018). In particular, glycosylation of S34 and S39 promotes the assembly of VIFs while S49 eliminates crosslinking of adjacent filaments; these processes contribute to the alterations of normal filament morphology. In mammalian cells, site-specific glycosylation of vimentin is required for the cytoskeletal modifications involved in cell migration (Tarbet et al., 2018).

## Role of Vimentin in Tractional Force Generation and ECM Contraction

As described in the discussion of PTMs above, some of the mechanisms by which vimentin regulate cell migration through ECM are now being defined. As vimentin interacts with actin filaments and microtubules to affect their structure and function (Mendez et al., 2010; Hookway et al., 2015), these interactions are also likely to regulate cell motility and the ability of migrating cells to remodel the ECM (Battaglia et al., 2018). Many types of cell migration rely on the formation and extension of relatively short (< 2 μm) membrane protrusions like filopodia, which then coalesce and contribute to the generation of lamellipodia and invadopodia (Lorenz et al., 2004; Yamaguchi et al., 2005; Baldassarre et al., 2006; Gardel et al., 2010). These protrusive regions of the cell are filled with highly polarized arrays of actin filaments situated in the cortex that enable the formation of longer (>10 μm) cell extensions. Cortical actin filaments are mechanically integrated with collagen fibrils by integrin receptors during early phases of cell migration and in collagen contraction (Grinnell et al., 2006; Ostrowska-Podhorodecka et al., 2021). In these processes, vimentin facilitates and supports cell extension formation through direct associations with actin filaments (Esue et al., 2006; Battaglia et al., 2018) or indirectly through interactions with proteins such as CARMIL2 (Lanier et al., 2015; Battaglia et al., 2018).

Vimentin is essential for the elongation of invadopodia (Yoneyama et al., 2014), and promotes lamellipodia growth and the formation and stabilization of long cytoplasmic extensions (Ding et al., 2020; Ostrowska-Podhorodecka et al., 2021). Moreover, vimentin colocalizes with non-muscle myosin II (Menko et al., 2014) and regulates the contractility of actomyosin bundles through the guanine exchange factor H1 and RhoA, which affect cell migration (Jiu et al., 2017) and tractional remodeling of the ECM. In fibroblasts, mechanosensitive actin stress fibers (Skau et al., 2018) along with vimentin filaments insert into ECM adhesions and regulate the mechanical integrity of cells and tissues (Costigliola et al., 2017).

## Impact of Vimentin on Single Cell and Collective Cell Migration

**FIGURE 4:**
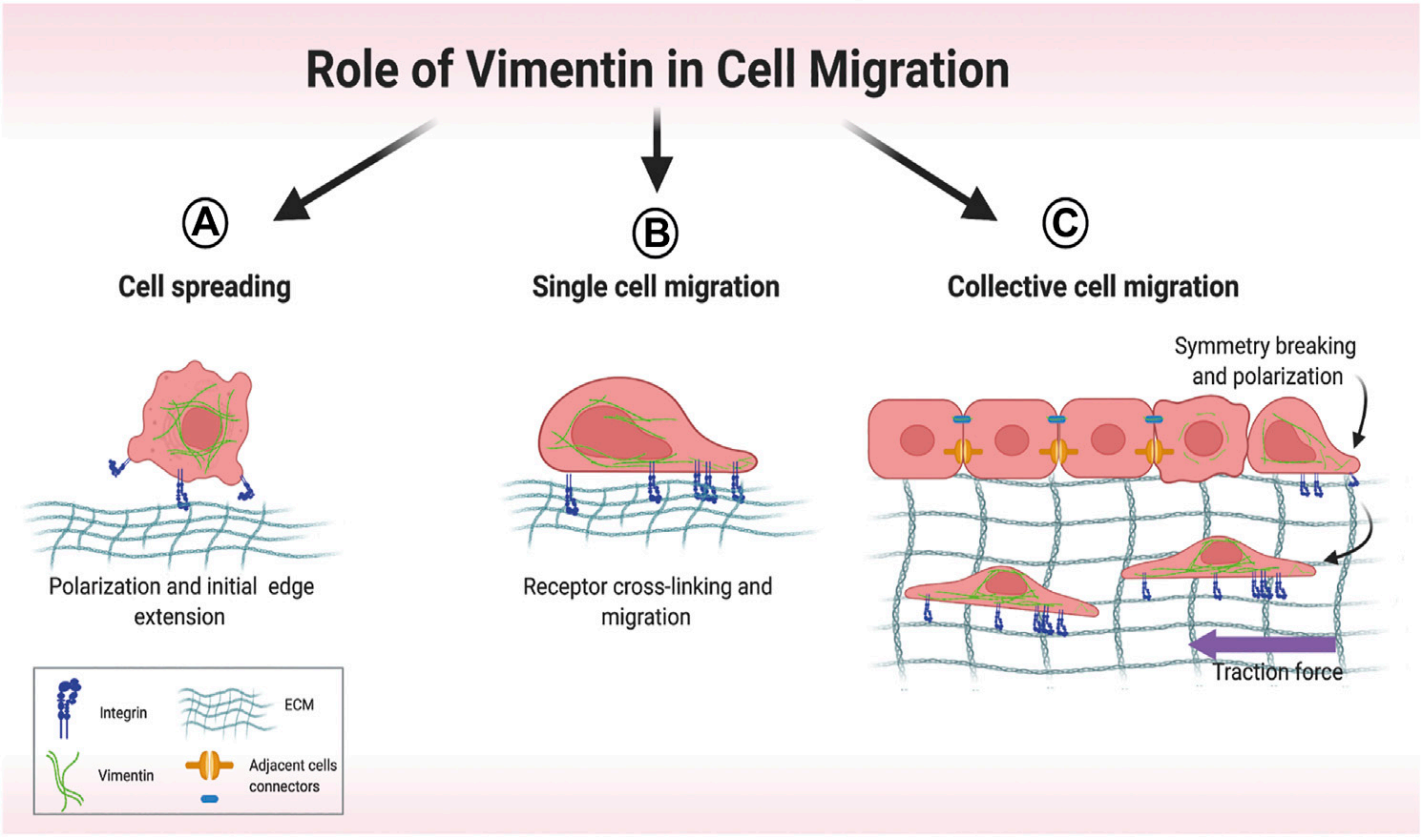
Overview of different modes of cell migration. **(A)** Early stages of cell spreading. Cell attachment to the extracellular matrix (ECM) involves initial cell polarization and edge extension. **(B)** Single-cell migration relies on vimentin-dependent cross-linking of collagen receptors. **(C)** In collective migration, cells are tightly connected, which restricts migration. Symmetry-breaking events lead to cell polarization and directed migration. Motile cells at the front exert attractive forces on their neighbors, which then coordinate their movement. Created with BioRender.com.

Arising from its involvement in several discrete cellular processes, VIF networks orchestrate cell spreading (Figure 4A) (Ostrowska-Podhorodecka and McCulloch, 2021), single cell migration (Figure 4B) (Patteson et al., 2019) and collective migration (Figure 4C) (De Pascalis et al., 2018). In cell spreading, the expansion of cell area on an unoccupied surface initiates adhesive interactions that enable cells to migrate (Janmey et al., 2021), which requires the formation of cell protrusions stabilized by VIFs (Ostrowska-Podhorodecka et al., 2021). Cell migration is strongly influenced by the nature of the local microenvironment. In two-dimensional single cell migration, cells spread within a single plane, which is guided by vimentin filaments (Ding et al., 2020). Vimentin mediates the transition of mesenchymal leader cells to a myofibroblast phenotype in the single cell migration of EMT (Walker et al., 2018). VIFs are also prominent elements of reparative cells at the wound edge and are associated with accelerated wound closure (Helfand et al., 2011; Menko et al., 2014).

At higher resolution, long (>4 μm) vimentin filaments serve as a load-bearing scaffolds to distribute traction stress during single cell migration (Costigliola et al., 2017). As wound closure involves the generation of contractile forces that can affect the structure of actin cytoskeletons, the vimentin network serves to diminish cell deformation (Janmey et al., 1991) and preserve cell integrity. Reduced vimentin expression in human mesenchymal stem cells is associated with increased deformation of the cell body after stretching (Sharma et al., 2018). Overexpression of vimentin in ameboid cancer cells contributes to cell resilience by limiting deformations in response to fast contractions (Lavenus et al., 2020), indicating that cell type-specific expression levels of vimentin endow cells with a broad range of mechanical properties.

Collective cell migration is promoted by the elevation of traction forces in the migrating cell monolayer and by the preservation of intercellular contacts (Figure 4C) (De Pascalis et al., 2018), which also serve to coordinate cell movement through dense connective tissue (Messica et al., 2017). In smooth muscle cells, VIFs are involved in the formation of intercellular junctions by associating with linker proteins such as plakoglobin and desmoplakin, which in turn connect with the cytoplasmic tails of cadherins (Delva et al., 2009; Tang et al., 2019). At the same time vimentin associates with actin filaments to facilitate intracellular and intercellular mechanical force transmission, which is required for cell motility (Tang, 2008).

Vimentin promotes collective cell migration by restricting actin flow, aligning tractional stress (Jiu et al., 2015; Costigliola et al., 2017; Battaglia et al., 2018) and supporting lateral cell–cell contacts (Mayor and Etienne-Manneville, 2016; Battaglia et al., 2018). In the collective cell migration that is seen in certain wound healing sites, reduced vimentin expression (along with nestin) diminishes the abundance of actin stress fibers parallel to the wound and promotes stress fiber formation perpendicular to the wound; these changes impact retrograde actin flow and the ability of cells to translocate (De Pascalis et al., 2018). In epithelial cells that express vimentin, VIFs are a component of junctional complexes that couple VE-cadherin to actin filaments, the IF cytoskeleton (Kowalczyk et al., 1998) and to FAs (Tsuruta and Jones, 2003; Kreis et al., 2005; Terriac et al., 2017), where VIFs bind to integrin-enriched matrix adhesions (Ivaska et al., 2007).

In highly motile epithelial cells, VIFs often co-distribute with keratin, the IF type first expressed in embryogenesis (Franz et al., 1983). In collective cell migration, co-expressed keratin and vimentin filaments exhibit spatially-distinct arrays (Osborn et al., 1980), which are necessary for preserving cytoplasmic viscoelasticity and for coupling through integrins to the ECM and to neighboring cells (Bilandzic et al., 2019; Yoon and Leube, 2019).

## Impact of Vimentin on Signaling Pathways That Affect Cell Migration and ECM

**FIGURE 5:**
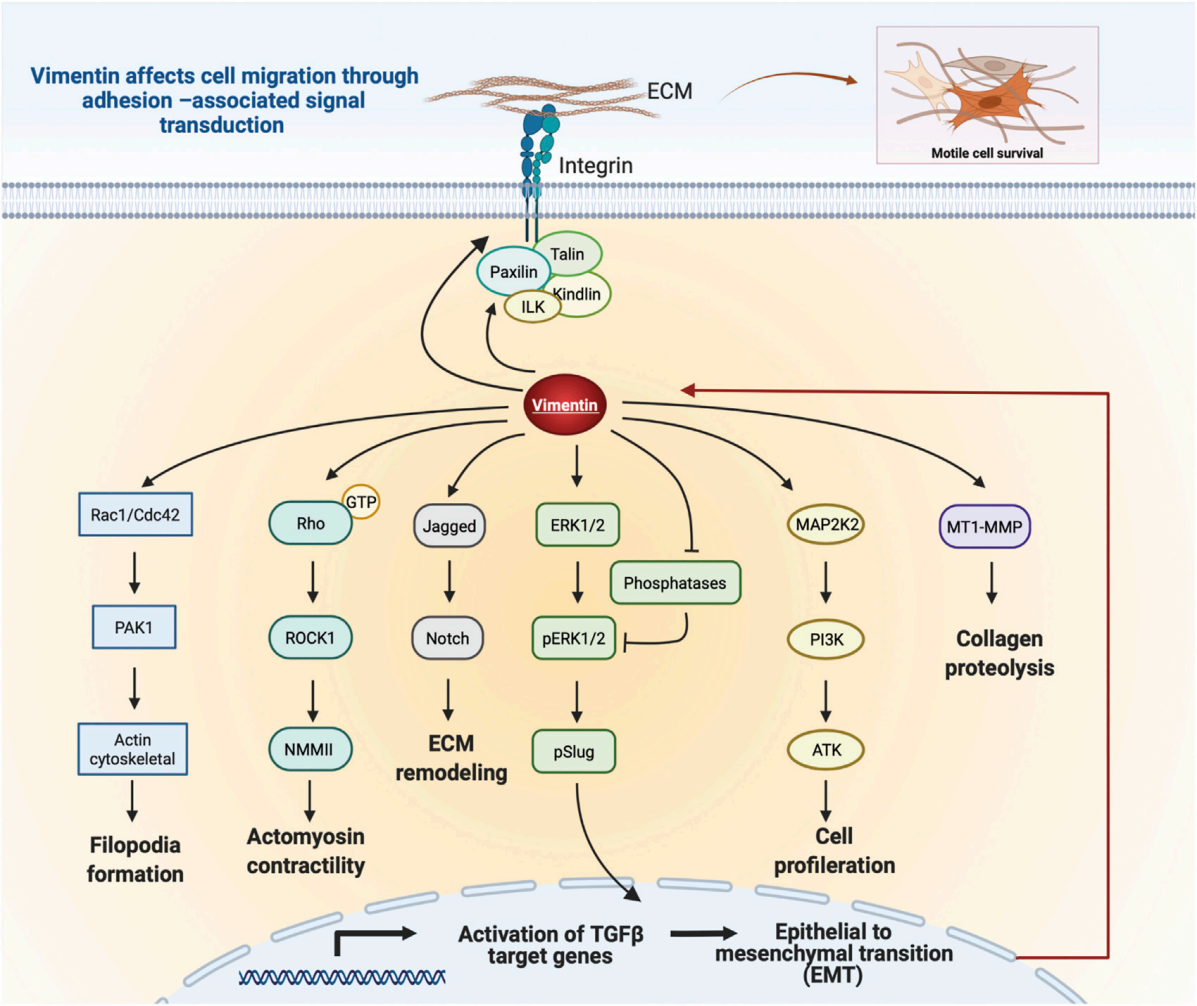
Schematic illustration of vimentin-dependent regulation of migratory signaling transduction. Vimentin coordinates filopodia formation by controlling actin filaments assembly via Rac1/Cdc42 and PAK1 pathways. Vimentin-dependent Rho/ROCK1 signalling transduction controls cell contractility and migration. Vimentin impacts Notch signaling by binding to Jagged1. Regulation of Slug phosphorylation and activity by Vim-ERK cooperation. Vimentin protects ERK from dephosphorylation and thus supports its activity and Slug phosphorylation. Vimentin coordinates cell proliferation by downregulation of the PI3K/AKT signalling cascade. Vimentin induces ECM remodeling by MT1-MMP-dependent collagen proteolysis. Vimentin filaments mediate integrin mechanotransduction and control the assembly of focal adhesions. Figure was created with BioRender.com.

Vimentin expression accelerates cell migration through paxillin-dependent regulation of Cdc42 activation, which leads to PAK1-dependent vimentin phosphorylation and filament assembly (Ostrowska-Podhorodecka et al., 2021). Thus, vimentin can regulate cell migration through its physical properties and by activating downstream signaling pathways that regulate cell movement (Figure 5). Further, vimentin affects Notch signaling in response to hemodynamic stress in the arterial wall. As a result of increased shear stress, phosphorylation of vimentin S38 initiates interactions between vimentin and Jagged1, which strengthens the Notch activation potential and adversely affects arterial wall remodeling and the ECM (van Engeland et al., 2019). Moreover, loss of vimentin in hepatic stellate cells decreases the phosphorylation of extracellular-signal regulated kinase (ERK) and AKT signaling, indicating a role for vimentin in controlling migration of hepatic stellate cells via the ERK/AKT and Rho pathways (Wang et al., 2019). Further, vimentin plays a role in wound healing through its regulation of TGF-β1 and Slug signalling, which are of central importance for ECM synthesis and remodeling and which manifest as the effect of vimentin depletion on suppression of the TGF-Slug–EMT pathway in fibroblasts (Cheng et al., 2016; Cheng and Eriksson, 2017). Taken together, these studies suggest an unexpected signaling role for vimentin in ECM structure and cell migration.

## Role of Vimentin in Regulating Integrin-dependent Cell Migration

Cells organize their migratory activities partly through tightly controlled protein-protein interactions in cell adhesions to the ECM. Integrins are transmembrane receptors expressed by many cells and are obligate heterodimers comprised of α and β subunits. The β subunit contains a large extracellular domain, a transmembrane domain and a cytoplasmic domain, while the α subunit forms a similar structure, but contains different motifs within its extracellular domain (Takada et al., 2007). The interaction of different α and β extracellular domains and their ability to bind to specific sequences, contribute to the ligand specificity exhibited by integrins. Intracellular signals that affect the cytoplasmic face of α and β subunits promotes allosteric modifications of integrins that affect their binding to extracellular ECM ligands. In this context, vimentin spatially localises to cell-matrix adhesions in various cell types (Danielsson et al., 2018) where vimentin filaments directly interact with β1 integrin (Kreis et al., 2005) and β3 integrin tails (Vohnoutka et al., 2019). Vimentin thus plays an essential role in the assembly and function of FA complexes.

Vimentin incorporates into nascent focal complexes and mature adhesions in a structure-dependent manner. While small vimentin oligomers (e.g., unit length filaments) are abundant in nascent adhesions, mature adhesions exhibit fully organized vimentin filaments (Terriac et al., 2017). In fibroblasts, vimentin deletion results in the limitation of the size of β1 integrin-rich focal adhesion through the inhibition of paxillin enrichment in adhesion sites (Terriac et al., 2017; Ostrowska-Podhorodecka et al., 2021). Further, vimentin is required for β1 integrin trafficking to the leading edge of migrating prostate cancer cells (Hafeez et al., 2011). Through its interactions with focal adhesion proteins like talin, the cytoplasmic domain of the β integrin subunit can interact with the actomyosin contractile machinery (Calderwood et al., 2013; Zacharchenko et al., 2016; Wang et al., 2019). In this context, vimentin plays an important role in the turnover of FAs and in the formation and release of integrin endocytic vesicles. Further, vimentin may regulate collagen remodeling through a functional link between β1 integrin and the membrane-bound collagenase, MT1-MMP (Galvez et al., 2002; Kwak et al., 2012). Vimentin complexes with the cytoplasmic tail of MT1-MMP and is necessary for MT1-MMP translocation to the plasma membrane (Kwak et al., 2012). Thus, vimentin influences the subcellular localization and activity of MT1-MMP, which through its interactions with the β1 and αvβ3 integrins, facilitates collective cell migration through collagen matrices (Galvez et al., 2002).

As vimentin can also be detected on the surface of cells (extracellular vimentin-ECV) and in the ECM after its release from activated macrophages (Mor-Vaknin et al., 2003; Frescas et al., 2017), there has been increasing interest in ECV and its potential effects on cell migration, particularly through modifications of the interactions of cell with the ECM through integrins. While ECV enhances axonal growth in injured mouse spinal cord (Shigyo and Tohda, 2016), the impact of ECV on cell motility and the ECM is not defined. Recent data indicate that ECV facilitates adherence of vimentin-negative MCF-7 cells to their underlying substrate. Further, gap closure and Transwell migration assays show that the migration rates of MCF-7 and MCF-10a cells are increased after treatment with ECV (100 ng/ml) (Thalla et al., 2021). The very limited data on the impact of ECV on cell migration and ECM suggest productive avenues for future research.

## Conclusion and Future Perspectives: A Role for Vimentin as a Regulator of Cell-Matrix Adhesions and Matrix Remodeling

In the context of ECM remodeling and cell migration, we considered how vimentin helps cells to sense, integrate, and respond to microenvironmental information. Vimentin can directly interact with focal adhesions and regulate cellular processes and discrete signaling pathways. These events in turn affect cell migration and ECM remodeling by spatially and temporally integrating arrays of external and internal signals. Cell-specific levels of vimentin expression, PTMs, and direct and indirect interactions with other cytoskeletal components, control the mechanical properties of cells during migration and ECM remodeling. It appears that the extraordinarily broad array of PTMs of vimentin and its structure in response to extracellular and intracellular mechanical signals are crucial for cell and tissue integrity (Costigliola et al., 2017; Messica et al., 2017; van Bodegraven and Etienne-Manneville, 2021). Expression of VIF may thus control the mechanical properties of the cells and modify their ability to remodel collagen by its incorporation as an adaptor protein in the β1 integrin adhesive machinery (Terriac et al., 2017; Ostrowska-Podhorodecka et al., 2021). Further studies on vimentin’s role in regulating cell signaling and matrix remodeling could advance our understanding of the pathology of vimentin-dependent changes in ECM remodeling, which contribute to the fibrosis associated with chronic inflammation. Further, a deeper knowledge of vimentin-dependent signaling systems in wound healing and tissue regeneration could provide new avenues for identifying drug targets for fibrosis and wound care.

## Abbreviations

VIF, Vimentin intermediate filaments; EMT, epithelial-mesenchymal transition; ECM, extracellular matrix; PTM, post-translational modifications; FA, focal adhesion; ECV, extracellular vimentin.
